# Cerebrovascular Events in Systemic Lupus Erythematosus: Diagnosis and Management

**DOI:** 10.31138/mjr.30.1.7

**Published:** 2019-03-28

**Authors:** Dionysis Nikolopoulos, Antonis Fanouriakis, Dimitrios T. Boumpas

**Affiliations:** 1 Rheumatology and Clinical Immunology Unit, 4th Department of Medicine, “Attikon” University Hospital, Athens, Greece,; 2 Laboratory of Immune Regulation and Tolerance, Autoimmunity and Inflammation, Biomedical Research Foundation of the Academy of Athens, Athens, Greece,; 3 Medical School, University of Cyprus, Nicosia, Cyprus.

**Keywords:** Cerebrovascular Events, Systemic Lupus Erythematosus, Brain Imaging, Drug Therapy

## Abstract

Stroke is a major cause of morbidity, mortality and disability in systemic lupus erythematosus (SLE). Patients with SLE have a two-fold increase in the risk of stroke with younger patients (ie, less than 50 years of age) having an ever-higher risk (up to 10-fold). Although the prognosis of SLE has improved, mortality due to cerebrovascular events (CVE) remains unchanged. Cerebrovascular disease may be directly attributed to the disease per se, as a manifestation of neuropsychiatric SLE, or be the result of traditional cardiovascular risk factors accompanying the disease. Elucidation of the underlying mechanism(s) of CVE is essential as it may guide the type of therapy (ie, antithrombotic or anticoagulant therapy versus immunosuppressive). Strokes attributed to lupus usually occur early in the course of the disease and are often accompanied by evidence of activity in other organs; those related to antiphospholipid antibodies can occur at any time, in patients with either active or inactive SLE. In this review, we discuss the epidemiology, work-up, management and primary prevention of CVE in patients with lupus. In view of the effectiveness of thrombolysis, physicians need to educate lupus patients and their families for the early recognition of the signs of stroke and the need to seek prompt attention. To this end acronyms, such as FAST (Facial drooping, Arm weakness, Speech difficulties and Time to call emergency service) can be used as a mnemonic to help detect and enhance responsiveness to the needs of a person having a stroke.

## INTRODUCTION

Neuropsychiatric systemic lupus erythematosus (NPSLE) may involve the central and peripheral nervous system in patients with SLE.^1^ The range of neuropsychiatric (NP) manifestations varies from serious events, such as myelitis, seizures and stroke, to more subtle events such as headache, mood disorders and mild cognitive dysfunction.^2^ Approximately 40% of patients experience some type of neuropsychiatric manifestations, with less than half of them directly attributed to SLE (*primary NPSLE*).^3^ The gold standard of NPSLE diagnosis remains physician judgment. Risk factors for NPSLE are prior major NP manifestation, generalized disease activity, and anti-phospholipid antibody (aPL) positivity.^4,5^

Cerebrovascular events (CVE) represent one of the most common and severe NP manifestations.^6^ Their prevalence varies from 3% to 20%, accounting for up to 15% of deaths in SLE.^7,8^ Strokes attributed to SLE tend to occur close to disease diagnosis and may be explained by systemic inflammation, endothelial activation or a prothrombotic state due to aPL.^9,10^ On the other hand, strokes unrelated to the disease usually occur at late stages and are caused by atherosclerosis due to traditional cardiovascular risk factors such as hypertension, hyperlipidemia and diabetes mellitus, which represent common comorbidities in SLE.^11^ Attribution of a CVE to SLE remains a challenge because, in the former case, immunosuppressive therapy should be considered.^4^

Herein, we review the epidemiology and discuss the issue of attribution of CVEs in SLE. We also propose a work-up in presenting with CVE and provide recommendations for primary prevention and treatment.

## EPIDEMIOLOGY

The majority of strokes related to SLE occur within the first year after diagnosis;^9^ thus the true prevalence of primary CV events can be captured even by epidemiological studies of short duration of follow-up. Strokes related to atherosclerosis and traditional factors tend to appear in late stages and thus need longitudinal cohort studies with prolonged follow-up.

The prevalence of stroke in SLE ranges from a little over 2% to as high as 19%,^6,9,12–15^ while the incidence ranges from 5.8 to 25.3 new cases per 1000 person-years.^6,9,12,13,15^ The large discrepancies between studies can be explained by various factors, such as duration of follow-up, race, age, and study design. A recent meta-analysis^16^ found a two-fold increased risk of ischemic stroke in SLE subjects compared to the general population, together with a three-fold increased risk of intracerebral haemorrhage, although individual studies have questioned the increased risk of intracranial hemorrhage.^12,13^ The relative risk is higher in younger ages,^17^ as young patients with SLE (age < 50 years) are almost 10 times more prone to develop CVE compared to subjects of similar age without SLE.^17^ The epidemiology of stroke also shows racial/ethnic as well as socioeconomic variations.^13,18^ An elevated relative risk of stroke has been observed among Hispanics and blacks compared to whites (11% and 36%, respectively). Low educational level and income is also associated with increased frequency of traditional cardiovascular factors and cardiovascular events in SLE patients.^18^ Shaharir et al.^19^ reported that males manifest cardiovascular events (including CVE) more frequently than females. A detailed presentation of epidemiologic data is depicted in *Table 1*. Of note, although survival of SLE patients has improved over the last decades,^20^ mortality rates due to complications of CVE have not decreased accordingly.^21^

**Table 1.** Studies reporting epidemiology of CVE in patients with SLE.

**Study**	**Incidence (1000pt/yrs)**	**Prevalence**	**RR**	**Median follow-up (years)**	**Comments**
Mok et al.^12^	6.45	4.08%	2.02	8	Duration of hospitalization and mortality rate similar between SLE and non-SLE patients.
Hanly et al.^6^	5.8	4.49%	NR	5.6	Fourth most frequent NP event in SLE, the majority attributable to lupus
Barbhaiya et al.^13^	5.88	2.19%	NR	3.7	Increased stroke risk among Blacks and Hispanics compared to Caucasians
Arkema et al.^9^	7.70	3.71%	2.2	NA	aPL carriers excluded
Chiu et al.^14^	NR	2.22%	1.67	7	11,637 newly diagnosed SLE patients and 58,185 subjects without SLE, matched for age, gender, and comorbidities (very large cohort)
Mikdashi et al. al.^15^	25.3	19%	NR	8	Higher prevalence and incidence compared to other studies, but small sample size (n= 238)

RR: Relative risk compared to the general population; NR: Not reported; NP: Neuropsychiatric; NA: Not applicable; aPL: Antiphospholipid antibodies.

In our experience from two lupus cohorts in Athens and Heraklion, Greece,^22,23^ 50 patients from a total of 1331 patients have suffered ischemic stroke, corresponding to an overall prevalence of 3.75%. Of them, 26 patients (52%) developed stroke in the context of secondary APS with only two having documented central nervous system (CNS) vasculitis (4%) (*Table 2*).

**Table 2.** Characteristics of CVE in SLE patients in “Attikon” and “Leto” SLE cohorts (n=1331).

**Diagnostic work-up**	

Number of patients with stroke	50

Ischemic stroke, n (%)	50 (100)

APS-related stroke, n (%)	26 (52)

CNS vasculitis, n (%)	2 (4)

Generalized lupus activity at the time of stroke, n (%)	30 (60)

**Treatment**	

Antiplatelets, n (%)	50 (100)

Anticoagulation, n (%)	17 (34%, all with APS)

Immunosuppressive Therapy, n (%)	24 (48)
✓ Cyclophosphamide, n (%)	✓ 17 (70.8%)
✓ Azathioprine, n (%)	✓ 7 (29.2)

## CLINICAL PRESENTATION OF STROKE

Acute stroke should be considered in any patient with SLE presenting with sudden onset neurological deficit and/or alteration in level of consciousness. Clinical presentation varies based on the type of stroke and the arterial territory involved. Typical symptoms include, but are not limited to: abrupt onset of hemiparesis or monoparesis, hemisensory deficits, visual disturbances, dysarthria, facial droop, ataxia, aphasia and sudden decrease in level of consciousness. These symptoms may occur isolated or, more likely, in various combinations. Differentiation between ischemic and haemorrhagic stroke is not possible on the basis of history/physical examination, although headache, vomiting, nausea, and abrupt loss of consciousness are more commonly observed in haemorrhagic strokes, highlighting the vital role of emergency brain imaging.

### Stroke as Initial Manifestation of Lupus

Stroke may be the presenting manifestation of lupus, and patients with SLE may suffer a stroke at a young age. Albeit rare, CVEs due to lupus in patients below 50 are common cause of referral to stroke units.^24^ A hospital-based study showed that the yearly incidence is 2.4 per 100.000 people (aged 20–24), 4.5 (aged 30–34) and 32.9 (aged 45–49).^25^

In a patient presenting with stroke, several conditions need to be sought for and ruled out. Extracranial arterial disease, mainly carotid dissection, but also vasculitis and other vasculopathies, may present with stroke. In such cases, contrast-enhanced magnetic resonance angiography (MRA) combined with carotid Doppler are recommended for the identification of carotid dissection.^26,27^ MRA with fat suppression is the best modality for carotid dissection if ultrasound is negative, while catheter angiography should be used in case of high suspicion of vasculitis or rare vasculopathies.^24^ Cardioembolism is another major cause that should be ruled out. *Patent foramen ovale* is one of the most common sources of unexplained stroke in young adults, thus transoesophageal echocardiography is recommended in every young patient without known cause of CVA. Holter electrocardiograms are also recommended as part of the initial work-up.^28,29^ Screening for hereditary thrombophilic disorders is not routinely recommended, as the latter are mainly associated with venous thromboembolic events. Less frequent causes of strokes are summarized in *Table 3*.

**Table 3.** Most common causes of stroke in patients < 50 years old.

**Causes of strokes in young patients**
***More common***
Cryptogenic (up to 30%)
Congenital and acquired heart disease
Drugs (Cocaine, methamphetamine)
***Less common***
Endocarditis
Arterial dissection
Traumatic
Fibromuscular dysplasia
CNS vasculitis
Large vessel vasculitis
Pregnancy
Oral contraceptives
Inherited prothrombotic states
SLE/APS
Systemic autoimmune diseases
Leukaemia and other malignancies
Metabolic disorders
Protein-losing enteropathy
Nephrotic syndrome

Among autoimmune diseases, the main diagnosis that should be considered in young patients presenting with stroke are SLE and/or APS. Young patients with unknown cause of stroke should be screened for antinuclear antibodies (ANA) and aPL (anti-cardiolipin [aCL], anti β2-glycoprotein I [β2-GPI] and lupus anticoagulant [LA]). If positive, aPL should be repeated after 3 months to diagnose definite APS.^30^ A systematic review showed that aPL were detected in 17.2% (2–56%) of patients with stroke and 11.7% (2–45%) of transient ischemic attack (TIA); conversely, the presence of aPL increases the risk for stroke approximately 5-fold.^31^

### Stroke in a Patient with Established SLE – The Challenge of Attribution

**Figure 1.**
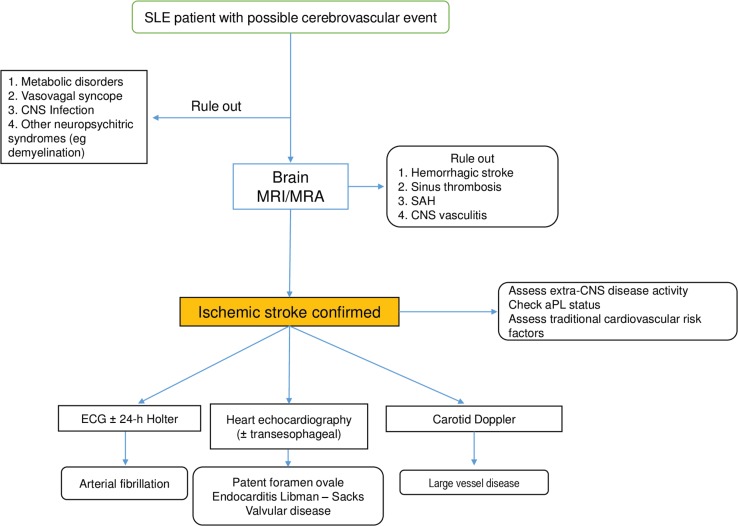
Initial work-up of an SLE patient presenting with stroke. SAH, subarachnoid haemorrhage; ECG, electrocardiogram; CNS, central nervous system; MRI/MRA, magnetic resonance imaging/angiography; aPL, antiphospholipid antibodies.

In a patient with known SLE, the physician should distinguish whether the stroke represents true primary NPSLE or is unrelated to the disease. Apart from the essential work-up outlined above, patients should be assessed for the presence of extra-neurological disease activity (eg, nephritis, arthritis, serositis, skin rashes, and hematologic abnormalities) (*Figure 1*). Use of a validated index of disease activity, such as the SLE Disease Activity Index (SLEDAI) is useful.^32^ Timing of CVE occurrence is also important, as most primary NP events tend to manifest near or early after the diagnosis of SLE. History of a previous major NP manifestation also constitutes a risk factor for primary NPSLE, and stroke in particular.^33^ Brain MRI/MRA is mandatory; the latter provides information for a possible vasculitic process, although documentation of frank vasculitis in SLE-related stroke is very rare in clinical practice. On the contrary, patients with long-standing lupus and presence of cardiovascular risk factors tend to experience strokes in the absence of generalized disease activity, due to atherosclerosis as a late sequel of the disease.^34^ The presence of sudden, severe headache in a lupus patient should raise the suspicion of cerebral sinus venous thrombosis (CSVT), subarachnoid haemorrhage (SAH) or CNS vasculitis.^35^

### White matter hyperintense lesions (WMHs) in SLE

Unidentified bright objects (UBOs) on MRI are hyperintense lesions seen on T2-weighted/fluid attenuated inversion recovery (FLAIR) images commonly identified in the subcortical white matter and other areas of the brain.^36^ These lesions are formed slowly due to vascular injury (*small vessel disease, SVD*) representing a “silent” process and are a common finding among older subjects. They have been proposed as a neuroimaging marker of *“brain frailty”* and have been associated with CV events and worse outcomes.^37^ The prevalence of these white matter hyperintensities (WMHs) increases with age and accrual of cardiovascular risk factors,^38–40^ but can also be seen in ∼5% of healthy young individuals.^41^

SVD is well recognized in SLE, confirmed also in autopsy studies,^42^ due to both inflammatory and non-inflammatory factors (small clots due to aPL, complement activation/immune complexes, plasma factors and endothelial cell adhesion molecules).^43^ WMHs have long been associated with NPSLE, being present in up to 60% of patients with CNS involvement, but with no correlation with a specific NP event.^44,45^ Approximately 18–40% of SLE patients *without any NP manifestation* also have such lesions on brain MRI.^46^ They are usually located subcortically and periventricularly in the frontal and parietal lobes (70–80%). Although there are no studies on association between WMHs and stroke in SLE, there are many studies on the clinical significance of WMHs in subjects without lupus.^36^ A meta-analysis of 22 longitudinal studies revealed a 3-fold increased risk of stroke in subjects with WMHs.^47^ Similarly, a large study in 1884 individuals with an average follow-up of 14.5 found that the relative risk for stroke and stroke-related mortality was 3.5 and 3, respectively, among subjects with WMHs ≤ 3mm; in those with both WMH ≥ 3mm and ≤ 3mm, the relative risk was 8.6 and 7, respectively. Patients with WMHs tend also to develop larger infracts with worse clinical outcomes.^48^ Although there are no studies on the management of asymptomatic WMH in SLE patients, physicians should consider these findings as evidence of cerebral vasculopathy and a risk factor for cerebrovascular disease; accordingly, an aggressive strategy against traditional cardiovascular factors should be adopted. Regarding immunosuppressive therapy, we do not routinely titrate immunosuppressive therapy, rather follow an individualized approach, based on number and size of lesions, as well as presence of symptoms. Finally, in asymptomatic patients, repeat MRI is not always warranted, but this should also be decided on a patient-by-patient basis.

## MANAGEMENT OF CVEs

### Primary prevention

Patients with SLE carry an increased risk of stroke, starting from the time of disease diagnosis, thus primary prevention is of utmost importance. Lupus-related risk factors include uncontrolled disease activity and aPL positivity.^4^ Moreover, SLE individuals with long-standing disease accumulate comorbidities, such as hypertension, diabetes mellitus and dyslipidaemia. When brain MRI is available, the presence of WMHs should be viewed as an additional risk factor. Smoking cessation is mandatory and modifiable cardiovascular risk factors should be treated appropriately, following a 10-year cardiovascular risk stratification using one of the available algorithms.^49^ Attainment of remission or -at least- low disease activity with immunosuppressive treatment should be the target of SLE therapy.^50^

Various imaging surrogate markers for atherosclerosis, including flow-mediated dilatation of the brachial artery, carotid intima media thickness and pulse wave velocity, have been tested in patients with SLE.^51^ Albeit these studies have added valuable information, no single surrogate marker (molecular or imaging) has yet shown a clear association with “hard” cardiovascular endpoints in lupus, ie, vascular events or cardiovascular mortality. Thus, outside the context of research studies, we do not routinely advise for screening with non-invasive modalities in our patients.

Although several studies have shown the beneficial effects of statins in the management of lupus,^52–56^ their use in SLE subjects with normal lipid status remains controversial. Two randomized controlled trials in a paediatric and an adult population failed to demonstrate a positive effect of statins on subclinical atherosclerosis progression over three and two years, respectively,^57, 58^ but follow-up may have been too short to detect an effect.

There is no clear recommendation for the use of aspirin as thrombophylactic agent in aPL-positive adults.^59^ Aspirin administration at low dose was recently recommended for all patients with juvenile onset lupus and positive aPL, by a recent initiative of a Task Force.^60^ In adults, a meta-analysis found a significant protective role of low-dose aspirin against thrombotic events in lupus patients with positive aPL.^61^ Thus, SLE patients with high-risk aPL profile (persistently positive medium/high titers or multiple positivity) may receive primary prophylaxis with antiplatelet agents, especially if other atherosclerotic/thrombophilic factors are present, after balancing for the bleeding hazard. In case of negative aPL, SLE patients may be candidates for preventative strategies as in the general population, including low-dose aspirin and/or lipid-lowering agents, based on their individual cardiovascular risk profile. A 10-year risk stratification should always be performed, although the risk may be underestimated in patients with lupus.^49^ In general, scepticism has recently been raised regarding the use of aspirin for primary prevention in high-risk populations (elderly and diabetics), after the publication of studies which showed that protection from cardiovascular events conferred by low-dose aspirin was largely counterbalanced by a significantly higher risk of major bleeding.^62,63^ However, one has to keep on mind that SLE patients represent a special group of individuals with chronic systemic inflammation and proven increased CVD risk.

Hydroxychloroquine (HCQ) is the cornerstone of lupus therapy due to its multiple beneficial effects.^64^ HCQ has additional antithrombotic actions; indeed, several prospective and retrospective studies have shown that HCQ reduces the relative risk of arterial thrombosis in general, and CVE, in particular, with hazard ratios ranging from 0.15 to 0.28 for cerebrovascular disease.^65,66,67^ Combination of HCQ with low-dose aspirin has been suggested to provide additive protection against stroke,^68^ but this needs to be further validated.

### Treatment and secondary prevention

**Figure 2.**
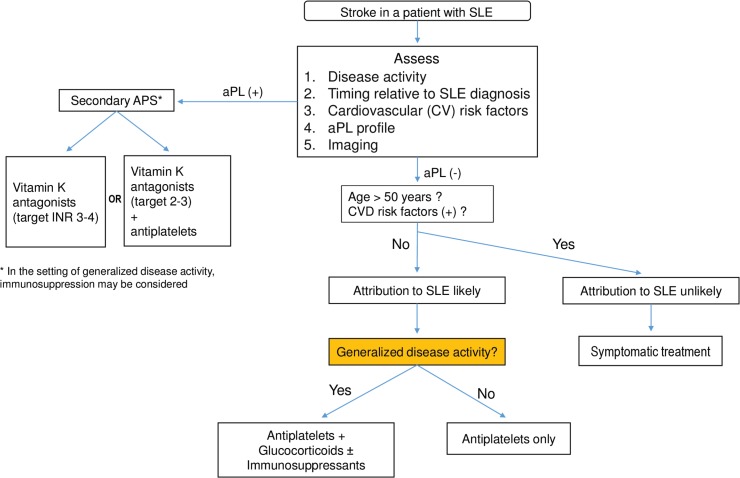
Management of stroke in SLE. APS, antiphospholipid syndrome; aPL, antiphospholipid antibodies; CVD, cardiovascular disease.

Treatment decisions in a SLE patient presenting with stroke should be based on whether the latter is considered directly related to the disease or not, and whether there is a coexisting APS or not. In the *acute phase*, the initial work-up and management does not differ between SLE patients and the general population.^4^ Symptomatic therapy similar to non-lupus patients should be provided in individuals with stroke occurring in the absence of generalized disease activity and/or APS. In particular, *thrombolysis* has been used successfully in lupus patients with stroke.^69,70^ In the setting of definite APS-related stroke, patients should be treated either with vitamin K antagonists with target INR 2–3 plus low-dose aspirin or vitamin K antagonists with target INR 3–4 long-term.^71^ In the first scenario, INR should be upgraded at 3–4, in case of stroke recurrence. Up to two-thirds of strokes in lupus occur in the setting of generalized disease activity,^23^ suggesting the role of systemic inflammation in the pathogenesis of CVE. In such cases, lupus-related stroke should be managed with high doses of glucocorticoids and/or major immunosuppressive therapy such as cyclophosphamide or rituximab, in order to suppress systemic inflammation.^5^ A recommended algorithm is depicted in *Figure 2.*

In our experience, 60% of CVE (30/50) occurred in the presence of generalized disease activity (SLEDAI≥6) and immunosuppressive therapy was instituted in 21/30 (70%). Specifically, 17 patients were treated with cyclophosphamide (CYC), 7 with azathioprine (AZA). Three patients were treated sequentially with AZA, CYC and finally with rituximab due to ongoing disease activity and severity of CVE. All patients (21/21, 100%) who received combined immunosuppression/antithrombotic treatment had a favourable outcome at 6 months and no recurrence was observed after at least three years of median follow-up (*Table 2*).

## CONCLUSIONS

Stroke represents one of the most devastating complications of lupus, as it is accompanied by significant disability, impaired quality of life and increased mortality. Over the last decades, the management of stroke in lupus has followed a “yin and yang” course, with all patients initially treated with high-dose glucocorticoids and/or cyclophosphamide, assuming that stroke was driven by vasculitis. With the recognition of the APS and the accelerated atherosclerosis in SLE, the balance tilted heavily towards anticoagulation and antiplatelet therapy. However, in more recent years, the realization that inflammation promotes thrombosis and the emergence of studies showing evidence for an increased frequency of stroke in the first years after the disease, has prompted discussions about combination therapy (anticoagulation and immunosuppression), especially in patients with generalized disease activity. WMHs, a common finding on brain MRI of SLE subjects, may represent an additional risk factor for CVE in lupus, but longitudinal studies are needed to confirm this hypothesis. In the meantime, increased vigilance and modification of atherosclerotic risk factors is essential. In view of the effectiveness of thrombolysis, physicians need to educate lupus patients and their families to the early recognition of the signs of stroke and the need to seek prompt attention. To this end the stroke association has proposed the *FAST* acronym used as a mnemonic to help detect and enhance responsiveness to the needs of a person having a stroke. The acronym stands for *Facial* drooping, *Arm* weakness, *S*peech difficulties and *T*ime to call emergency service.
